# Valorization of Aromatic Plant Distillation Residues: Phenolic Composition, Antioxidant Capacity, and Antimicrobial Activity of *Rhododendron tomentosum* Harmaja Extracts

**DOI:** 10.3390/molecules31101579

**Published:** 2026-05-09

**Authors:** Izabela Jasicka-Misiak, Halyna Kukhtenko, Yulian Konechnyi, Liudas Ivanauskas, Mindaugas Marksa, Ján Brindza, Oleksandr Kukhtenko

**Affiliations:** 1 Department of Pharmacy and Ecological Chemistry, Institute of Chemistry, University of Opole, 48 Oleska St., 45-052 Opole, Poland; 2 Department of Industrial Technology of Medicines and Cosmetics, National University of Pharmacy, 53 Hryhoriia Skovorody St., 61002 Kharkiv, Ukraine; 3 Department of Microbiology, Danylo Halytsky Lviv National Medical University, 69 Pekarska St., 79010 Lviv, Ukraine; 4 Department of Analytical and Toxicological Chemistry, Lithuanian University of Health Sciences, A. Mickeviciaus g. 9, LT-44307 Kaunas, Lithuania; 5 Institute of Plant and Environmental Sciences, Slovak University of Agriculture in Nitra, 2 Trieda Andreja Hlinku St., 949 76 Nitra, Slovakia

**Keywords:** *Rhododendron tomentosum* Harmaja, *Ledum palustre* L., by-products, valorization of residues, waste, GC-MS, HPLC-PDA, HPTLC

## Abstract

Hydrodistillation of aromatic plants for essential oil production generates substantial amounts of solid and liquid residues that are commonly discarded despite their potential value as sources of bioactive compounds. In this study, the essential oil and post-distillation residues of *Rhododendron tomentosum* Harmaja were evaluated within a waste-to-value framework to recover phenolic compounds with antioxidant and antimicrobial properties. Dry extracts obtained from liquid (DEA) and solid (DEE) residues were characterized in terms of total phenolic and flavonoid contents, antioxidant capacity (DPPH assays), and antimicrobial activity against selected microorganisms. Quantitative HPLC–PDA analysis revealed multiple phenolic compounds. Extracts derived from solid residues exhibited significantly higher phenolic and flavonoid contents and stronger antioxidant activity than those obtained from liquid residues, indicating that solid by-products constitute a richer phenolic matrix. Antimicrobial assays revealed pronounced activity for extracts prepared from plant material harvested in October, particularly those based on propylene glycol and glycerin, which were effective against both Gram-positive bacteria and selected Gram-negative clinical isolates. The essential oil showed broad-spectrum antimicrobial activity, including inhibition of *Aspergillus niger*. Stability studies demonstrated that the phenolic composition and bioactivity of the dry extracts were largely preserved after one year of storage. These findings demonstrate that *R. tomentosum* hydrodistillation residues represent a promising source of natural antioxidants and antimicrobial agents, supporting their potential utilization as value-added ingredients in food and cosmetic applications and contributing to circular economy strategies.

## 1. Introduction

Aromatic plants are naturally rich sources of essential oils, and their components have multifunctional potential applications in various fields, including medicine, cosmetics, and food [1,2]. In most aromatic plants, essential oil content does not exceed 1%, meaning that approximately 99% of the plant material remains as residue after hydrodistillation. These post-distilled residues, rich in secondary metabolites, may serve as valuable sources of bioactive compounds which have gained increasing interest in recent years [3,4,5,6]. A comprehensive review of recent scientific reports emphasizes that agro-industrial and food-processing by-products constitute an increasingly important source of polyphenol-rich extracts with potential applications as natural antioxidants, antimicrobials, and functional food ingredients. In particular, Bernini et al. critically analyzed phenolic compounds recovered from a wide range of agro-industrial residues, highlighting advances in green extraction strategies, structure–activity relationships, and the relevance of these compounds within circular economy frameworks [7]. Post-distillation residues, previously regarded as waste, are now being reconsidered as promising sources of phenolic compounds and other secondary metabolites. Several studies have demonstrated that such residues retain significant amounts of polyphenols, flavonoids, and phenolic acids. For example, solid residues obtained after the distillation of *Hyssopus officinalis* L., *Thymus mastichina* L., *Lavandula × intermedia* var. Super, and *Salvia lavandulifolia* Vahl. were analysed for their phenolic compound content and their activity against the spoilage mold *Penicillium verrucosum* Dierckx. [8]. Twenty three phenolic compounds were identified in the waste of lavandin (*Lavandula × intermedia* Emeric ex Loiseleur) [9]. By-products of *Lavandula latifolia* were shown to contain rosmarinic acid, apigenin, and luteolin [10]. Psarrou et al. analysed the post-distilled residues of rosemary and detected the presence of rosmarinic acid, carnosic acid, and carnosol in the recovered waste [11]. These findings highlight a shift from considering post-distillation residues as waste to recognizing them as valuable sources of secondary metabolites. Most studies emphasize the dual potential of these residues as sources of antioxidant and antimicrobial agents. For example, fourteen phenolic compounds were identified in the post-distillation residues of *Salvia aegyptiaca*, and their antioxidant properties were investigated [12]. Sánchez-Vioque et al. analyzed the polyphenols present in solid residues from the steam distillation of *Cistus ladanifer*, *Lavandula × intermedia*, *Santolina rosmarinifolia*, and *Thymus mastichina*, and evaluated their antioxidant activities [13]. Post-distillation extracts of anise and star anise demonstrated significant antibacterial activity and synergistic effects with conventional antibiotics against multidrug-resistant bacterial strains, highlighting their potential as cost-effective natural antimicrobial agents [14]. In addition to *Lamiaceae* species, plants from the *Ericaceae* family have also been investigated for the valorization of post-distillation by-products. For instance, *Arbutus unedo* L. residues were reported to possess high polyphenol content and strong antioxidant activity, comparable to vitamin C, along with the ability to reduce reactive oxygen species formation in human skin cells under oxidative stress [15].

Thus, recent advances in sustainable bioprocessing and the circular economy have highlighted the importance of waste valorization for converting by-products into value-added compounds [16,17,18]. The by-products of essential oil production, including hydrolats and dry solid residues, represent alternative sources of bioactive compounds. These materials can be exploited not only for their antioxidant potential but also for their antimicrobial activity against pathogenic and spoilage microorganisms [8,19].

Aromatic plants are widely recognized as rich sources of bioactive compounds with therapeutic, preservative, and industrial significance. *Rhododendron tomentosum* Harmaja (*Ericaceae* family), also known as *Ledum palustre* L., has traditionally been used in ethnomedicine for the treatment of various ailments, including rheumatism, coughs, colds, insect bites, and as a natural repellent [20,21,22]. *R. tomentosum* is a fragrant evergreen shrub that grows in peaty soils across northern Europe, Asia, and North America. It is commonly referred to as wild rosemary, marsh tea, marsh rosemary, or Labrador tea [23]. The essential oil of *R. tomentosum*, along with its polyphenol-rich fractions, has demonstrated analgesic, anti-inflammatory, antimicrobial, antiviral, antifungal, and insecticidal properties in both in vivo and in vitro studies [24]. In addition, recent scientific research has highlighted its promising antidiabetic and anticancer activities [25].

In this context, the present study aims to evaluate the post-distilled residues obtained after the hydrodistillation of *R. tomentosum* in terms of their phenolic content, antioxidant, and antimicrobial potential.

## 2. Results and Discussion

**Figure 1:**
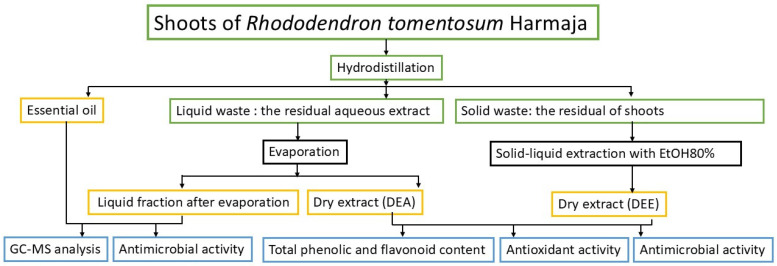
Flowchart of post-distillation residue analysis process.

Approximately 93% of total essential oil production is obtained by water distillation techniques; consequently, this method generates the largest quantity of distillation residues [5]. These residues can be classified into solid and liquid fractions (wastewater). In hydrodistillation, plant material is boiled in water, generating steam in situ, which facilitates the extraction of volatile compounds. At the same time, non-volatile constituents are dissolved in the aqueous phase. To recover these compounds, the wastewater was subjected to evaporation, resulting in two fractions: a dry extract (DEA) and a remaining liquid fraction. In parallel, the solid waste was macerated with 80% ethanol, and the resulting extract was evaporated to obtain a dry extract (DEE). Thus, hydrodistillation yielded four types of products: essential oil, a liquid fraction obtained after evaporation of wastewater, and two dry extracts (DEA, DEE) derived from plant material collected in July, August, September, and October. The essential oil and liquid fraction were analysed by GC–MS and tested for antimicrobial activity, while dry extracts were characterized by HPLC–PDA and evaluated for phenolic content (TPC, TFC), antioxidant capacity (DPPH), stability, and antimicrobial activity. For antimicrobial testing, dry extracts were dissolved in propylene glycol and glycerine. Thus, the applied hydrodistillation waste recycling strategy is illustrated in Figure 1.

Shoots of *R. tomentosum* were processed according to the procedure shown in Figure 1, and the yields of the obtained extracts are presented in Table 1.

The essential oil yield was 0.87 ± 0.09%. During hydrodistillation, partial extraction of water-soluble compounds into the boiling water occurred, resulting in a liquid fraction, which, upon evaporation, yielded a dry extract (DEA) with a yield of 17.11 ± 1.11% and a remaining liquid fraction (91.25 ± 2.21%). Re-extraction of the solid waste with 80% ethanol yielded a dry extract (DEE) with a yield of 5.05 ± 0.59%. The total extractable material amounted to approximately 22% of the dry plant, indicating a high recovery of compounds with potential biological activity in addition to the essential oil.

### 2.1. Chemical Profiles of Essential Oils and Liquid Fraction Obtained After Evaporation Wastewater

The essential oils of *R. tomentosum*, collected in different months, were mainly composed of monoterpenes. Monoterpene hydrocarbons made up 14.9–22.5%, while oxygenated monoterpenes accounted for 43.3–56.6% of the total oil. Sesquiterpenes were present in smaller amounts: 3.6–9.9% for sesquiterpene hydrocarbons and 12.7–25.3% for oxygenated sesquiterpenes. The chemical composition of the essential oils varied depending on the month of collection. In total, 49 compounds were identified in the essential oils, as listed in Table 2. All of the identified compounds have been previously found in *R. tomentosum* (*Ledum palustre* L.). Regardless of the collection period, the main components of the oils consistently included terpineol <γ-> (21.0–36.9%), palustrol (8.8–17.2%), ledol (3.6–8.1%), cymene <ρ-> (9.0–9.4%), geranyl acetate (5.4–5.8%), aromadendrene <allo-> (2.8–5.9%), bornyl acetate (4.3–4.6%), terpinen-4-ol (2.2–2.6%), and terpinene <α-> (1.3–4.2%). Compared with previously published data, the main components of the essential oil of *Ledum palustre* L. (*R. tomentosum*) growing in Estonia are terpineol <γ-> (RI 1238, 31.2%), palustrol (RI 1557, 15.9%), and ledol (RI 1594, 11.8%) [26]. The same three major compounds were described in another study, where terpineol <γ-> (RI 1238, 18.7–24.7%), palustrol (RI 1557, 16.7–23.9%), and ledol (RI 1594, 12.8–16.5%) [27]. Anna Jesionek et al. also reported these compounds as predominant in samples collected in Poland: terpineol <γ-> (RI 1245, 8.5–22.2%), palustrol (RI 1568, 0.8–13.0%), and ledol (RI 1603, 2.3–14.4%); in another report, terpineol <γ-> (RI 1245, 15.0–18.8%), palustrol (RI 1582, 11.5–15.7%), and ledol (RI 1603, 9.6–12.1%) [28,29]. At the same time, another group of researchers identified three major components of the essential oil of *R. tomentosum* grown in Lithuania: iso-ascaridole (RI 1303, 16.7–24.1%), palustrol (RI 1568, 11.9–31.8%), and ledol (RI 1602, 11.1–23.4%) [30]. Similarly, Sameh Baananou et al. reported ascaridole (RI 1238, 4.5–15.1%), palustrol (RI 1569, 41.0–43.4%), and ledol (RI 1602, 23.3–26.7%) as the main components in Lithuanian samples [31]. Another study from Lithuania listed palustrol (RI 1568, 21.0%), ascaridole (RI 1237, 4.0%), and lepalone (RI 1282, 3.0%) as dominant compounds [32]. G. Benelli et al. reported cis-ascaridole (RI 1233, 14.8 ± 2.2%), iso-ascaridole (RI 1301, 20.5 ± 1.9%), and cymene <ρ-> (RI 1022, 25.5 ± 3.4%) as major chemical constituents in *R. tomentosum* from Poland [33]. In contrast, a single dominant compound, ascaridole (RI 1245), was identified in plants grown in Canada, reaching 64.7 ± 0.7% [34].

The composition of the liquid fraction obtained after evaporation of the liquid waste is summarized in Table 3. In contrast to the essential oil, the volatile components of the liquid fraction are mainly represented by oxygenated monoterpenes and oxygenated sesquiterpenes, which together account for 26.9–39.9% of the identified compounds. A total of 24 volatile components were identified, some of which were also present in the essential oil, while others were unique to the liquid fraction. Compounds not detected in the essential oil include benzyl alcohol, cresol <ρ->, phenyl ethyl alcohol, sabina ketone <dehydro->, pinene oxide <β->, ascaridole, cymen-8-ol <ρ->, cryptone, piperitone epoxide <trans->, cymen-7-ol <ρ->, fenchol <2-ethyl-exo->, cyperotundone and 2,4,4,7-tetramethyl-5,7-octadien-3-ol. 2,4,4,7-Tetramethyl-5,7-octadien-3-ol accounts for 23.5–26.3% of the total composition of volatile compounds in the liquid fraction. The conducted studies demonstrated that the wastewater obtained after hydrodistillation of *R. tomentosum* contains oxygenated volatile compounds.

### 2.2. Polyphenolic Profiles of Solid and Liquid Wastes from Hydrodistillation

Residual extracts remaining after essential oil isolation are often regarded as waste. However, these extracts have significant potential as valuable by-products of the distillation process, being rich in polyphenolic compounds. Polyphenols are a diverse group of naturally occurring plant compounds synthesized as secondary metabolites during plant development, particularly in response to environmental stressors such as pathogens and ultraviolet radiation [37]. Based on their chemical structure, polyphenols are primarily classified into simple phenylpropanoids (including cinnamic acid and its derivatives such as p-coumaric, ferulic, caffeic, and sinapic acids), phenolic acids (hydroxycinnamic and hydroxybenzoic acids), flavonoids (flavones, flavonols, flavanones, anthocyanins, isoflavonoids, etc.), lignans and lignin, coumarins, and stilbenoids [38]. Due to their unique chemical structures and strong free radical scavenging capacity, the consumption of polyphenol-rich foods has been associated with numerous health benefits, including the prevention of cardiovascular diseases, cancer, and age-related disorders [39,40,41,42]. Given the high bioactive potential of polyphenolic compounds, it is essential to determine their content in dry extracts obtained from post-distillation residues, which may be further used in pharmaceutical formulations, dietary supplements, and for the fortification of food products [43,44,45].

**Figure 2:**
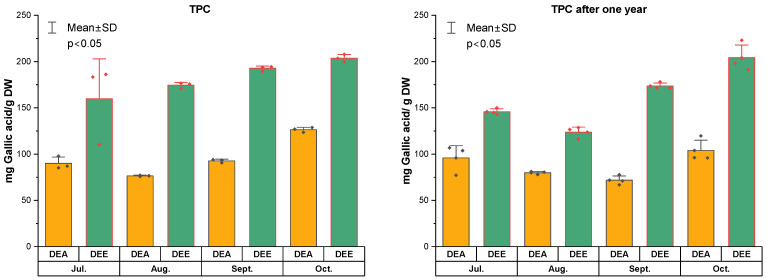
Total phenolic content (TPC) of dry extracts (DEA from liquid wastes and DEE from solid wastes) of *Rhododendron tomentosum* Harmaja obtained from plant material collected in July, August, September, and October, determined after extraction and after one year of storage.

**Figure 3:**
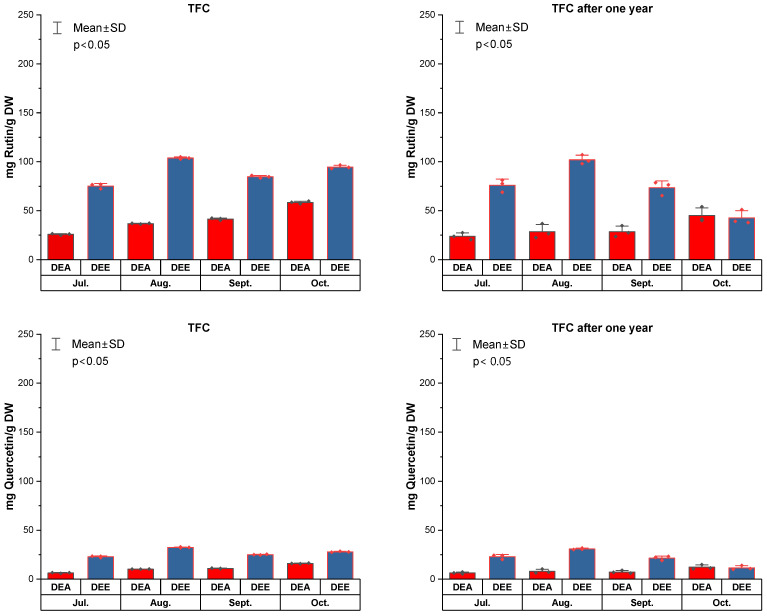
Total flavonoid content (TFC) of dry extracts (DEA from liquid wastes and DEE from solid wastes) of *Rhododendron tomentosum* Harmaja obtained from plant material collected in July, August, September, and October, determined after extraction and after one year of storage.

The TPC and TFC in the dry extracts DEA and DEE, obtained after hydrodistillation of *R. tomentosum* shoots, are presented in Figure 2 and Figure 3. Quality control analyses of polyphenol content were also performed after one year. Higher total phenolic content values of 159.9 ± 43.1 to 203.6 ± 4.1 mg GAE/g were observed in extracts obtained by 80% ethanol extraction of solid residues (DEE), whereas lower values of 76.5 ± 0.6 to 126.4 ± 2.6 mg GAE/g were found in extracts obtained by evaporation of liquid wastes (DEA). DEE extracts prepared from raw material harvested in September and October showed slightly higher TPC values. After one year of storage, follow-up quality-control analyses of polyphenol content were performed. TPC values of 123.7 ± 6.7 to 204.1 ± 16.6 mg GAE/g were observed for DEE extracts, while 71.8 ± 5.5 to 103.9 ± 13.6 mg GAE/g were found for DEA extracts. Overall, total polyphenol content decreased by approximately 5–20% over one year of storage.

The obtained results were compared with literature data on post-distillation residues from various essential oil-bearing plant species. Solid waste obtained after the essential oil distillation of *Lavandula latifolia* was reported to contain TPC ranging from 1.89 to 3.54 mg GAE/g dry weight [10,46]. By-products of *Salvia aegyptiaca* contained TPC levels between 69.49 and 88.41 mg GAE/g dry weight [12]. In *Thymus vulgaris*, TPC levels ranged from 17.5 to 34.5 mg GAE/g dry weight [47], and from 35.69 to 72.83 mg GAE/g dry weight in post-distillation waste materials [48].

Maria Irakli et al. analysed post-distilled residues from essential oil industry plants of the *Lamiaceae* family and reported the following TPC values: *Melissa officinalis* L. 100.53 mg GAE/g, *Mentha spicata* 99.76 mg GAE/g, *Origanum vulgare* L. 90.49 mg GAE/g, *Salvia fruticosa* Mill. 66.92 mg GAE/g, *Rosmarinus officinalis* L. 59.74 mg GAE/g, and *Satureja thymbra* L. 47.91 mg GAE/g dry weight [49]. These findings confirm that post-distillation plant materials retain significant levels of polyphenolic compounds, with values varying depending on the plant species. Branimir Pavlić et al. also reported high TPC values 90.20–290.28 mg GAE/g in dry extracts obtained from herbal dust of *Salvia officinalis* L., a by-product generated during filter tea production [50].

In DEA extracts from liquid waste, TFC ranged from 6.3 ± 0.3–16.1 ± 0.4 mg QE/g (TFC after one year 6.4 ± 0.9–12.2 ± 2.2 mg QE/g) and 25.8 ± 0.7–58.4 ± 1.3 mg RE/g (TFC after one year 23.8 ± 3.5–45.1 ± 7.7), while in DEE extracts from solid waste, TFC was higher–22.9 ± 0.8–32.4 ± 0.3 mg QE/g (11.6 ± 2.1–30.8 ± 0.8 mg QE/g) and 75.2 ± 2.5–103.7 ± 1.1 mg RE/g (42.6 ± 7.2–101.9 ± 4.6 mg RE/g) (Figure 3). The total flavonoid content in the studied extracts decreased by 2–15% over one year of storage. Analysing the results of the TFC determination presented in Figure 3, a significant difference (*p* < 0.05) in the quantitative content was observed between the dry extracts obtained from post-distillation solid and liquid residues.

As evident from the data, the choice of an appropriate compound for the spectrophotometric determination of TFC is of significant importance. The maximum absorption of quercetin occurs at 425 nm, whereas the maximum absorption of the analysed samples was observed in the range of 400–405 nm for DEA extracts and 410–414 nm for DEE extracts. The absorption maximum of rutin lies within the range of 407–410 nm, which closely corresponds to the absorption maxima observed for the studied samples. Therefore, the quantitative data on TFC expressed in terms of rutin can be considered valid. Given the potential discrepancies in TFC results depending on the reference standard used—as well as the relevance of method validation parameters such as linearity, regression coefficient—it is essential to present the corresponding absorption spectra. This issue was addressed in our previous work and has also been discussed by other researchers [24,51,52,53].

Comparing the TFC in the post-distillation residues of *R. tomentosum* shoots with published data reveals that *R. tomentosum* contains a notably higher amount of flavonoid compounds than many plants from the *Lamiaceae* family. For example, TFC values ranging from 59.4 to 94.3 mg QE/g were observed in *Ocimum basilicum* L. residues after enzyme treatment [54]. In *Thymus vulgaris* L. post-distillation waste, TFC ranged from 11.31 mg QE/g to 52.89 mg QE/g [48]. Additionally, spent extracts of *Origanum vulgare* L. exhibited a TFC of 34.88 mg RE/g, *Thymus vulgaris* L. showed 28.16 mg RE/g, and *Ocimum basilicum* L. contained 27.59 mg RE/g [55]. In contrast, *Foeniculum vulgare* seed residual after essential oil extraction had a significantly lower TFC of 5.22 ± 0.01 mg RE/g extract [56].

HPTLC analysis was performed to monitor possible compositional changes in the dry extracts DEA and DEE after preparation and after one year of storage (Supplementary Data, Figure S1a,b). Comparison of the HPTLC fingerprints revealed no differences in the overall profile. Moreover, comparison with previously published HPTLC fingerprint data for *R. tomentosum* liquid extracts showed no significant changes in the general phytochemical pattern, indicating good stability of the compounds after hydrodistillation [24].

**Figure 4:**
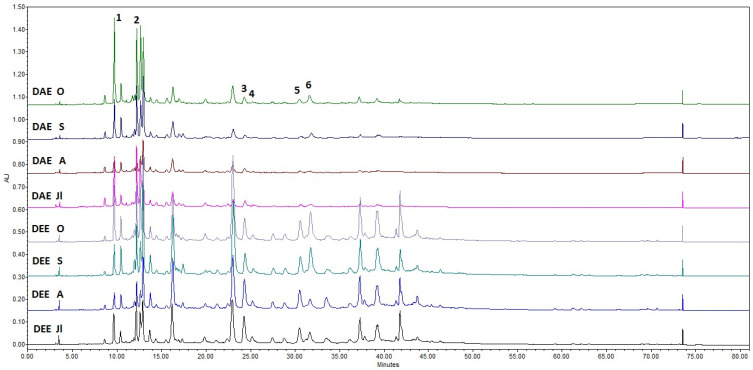
HPLC-PDA profiles at 325 nm showing the separation of phenolic compounds in the dry extracts DEA and DEE of *Rhododendron tomentosum* Harmaja. The peak numbers corresponding to Figure 4 are listed in Table 4.

The phenolic profiles of the dry extracts DEA and DEE, obtained after the hydrodistillation of *R. tomentosum* shoots and analysed by HPLC-PDA after one year of storage, are presented in Figure 4 and Table 4.

Neochlorogenic acid, chlorogenic acid, hyperoside, isoquercitrin, avicularin and quercitrin were detected in all dry extracts. Chlorogenic acid was identified as the predominant compound in the DEA extracts derived from liquid waste 1.15 ± 0.11 to 3.80 ± 0.51 mg/g. In the DEE extracts obtained from solid waste, its content was at a comparable level and ranged from 1.53 ± 0.12 to 3.12 ± 0.30 mg/g. The dominant component in the DEE extracts was hyperoside, with concentrations ranging from 5.40 ± 0.63 to 8.37 ± 0.96 mg/g, followed by isoquercitrin, which ranged from 1.48 ± 0.17 to 7.33 ± 0.86 mg/g. The identified compounds neochlorogenic acid and chlorogenic acid belong to the group of hydroxycinnamic acids, whereas hyperoside, isoquercitrin, quercitrin, and avicularin are classified as quercetin flavonol glycosides. These compound classes contribute significantly to their antioxidant and anti-inflammatory properties [57,58,59]. The obtained data indicate a trend of increased accumulation of secondary metabolites during the autumn season.

### 2.3. Antioxidant Activity

**Figure 5:**
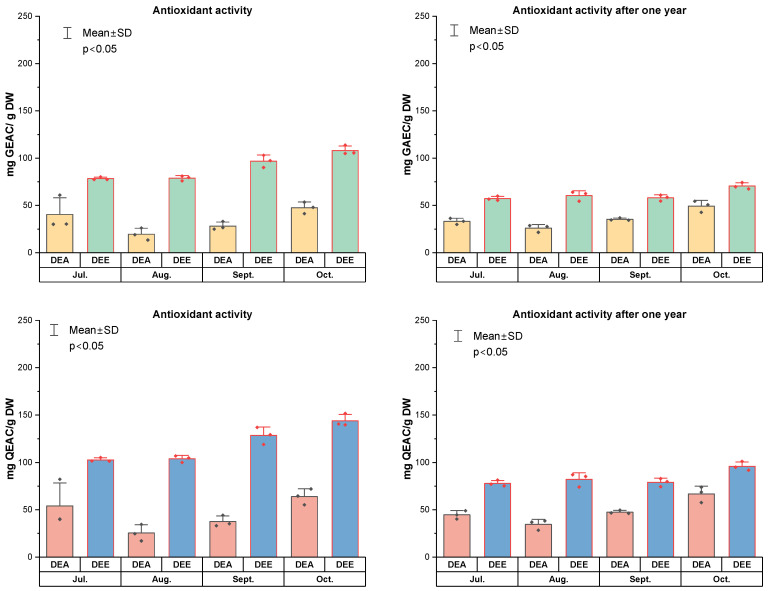
Antioxidant activity (DPPH) of dry extracts (DEA from liquid wastes and DEE from solid wastes) of *Rhododendron tomentosum* Harmaja obtained from plant material collected in July, August, September, and October, determined after extraction and after one year of storage.

Given that medicinal plants are recognized for their high antioxidant potential, it is important to evaluate the antioxidant capacity of dry extracts obtained as post-distillation residues [60]. The antioxidant activity of *R. tomentosum* dry extracts was determined using the DPPH assay. The results of the antioxidant activity are presented in Figure 5. These data illustrate the varying degrees of free-radical scavenging capacity. In DEA dry extracts, antioxidant activity ranged from 25.9 ± 3.8–49.2 ± 6.0 (after one year 19.5 ± 6.3 to 47.6 ± 6.1) mg GEAC/g and from 25.4 ± 8.7 to 63.9 ± 8.3 (after one year 34.5 ± 5.3–66.7 ± 8.2) mg QEAC/g. In contrast, DEE dry extracts exhibited higher antioxidant activity, ranging from 78.2 ± 1.5 to 108.1 ± 4.8 (after one year 57.2 ± 2.2–70.4 ± 3.3) mg GEAC/g and from 102.7 ± 2.1 to 143.8 ± 6.8 (after one year 77.8 ± 3.1–96.0 ± 4.6) mg QEAC/g. Antioxidant activity of DEA and DEE dry extracts was consistent with polyphenol content. These findings highlight the contribution of the complex polyphenolic matrix of the extracts to their antioxidant activity, even after heat treatment of the plant material. Numerous studies highlight the high antioxidant and biological potential of post-distillation by-products [61].

### 2.4. Antimicrobial Activity

**Figure 6:**
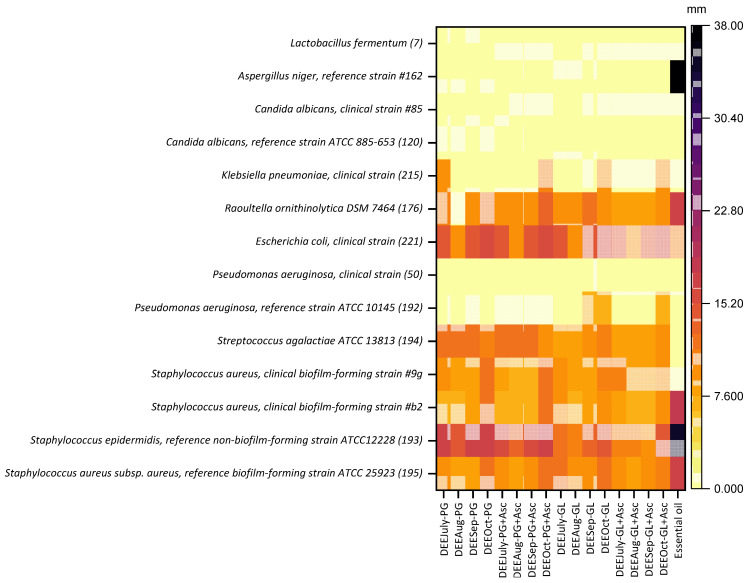
Antimicrobial activity of the dry extracts of *Rhododendron tomentosum* Harmaja and essential oil determined by the agar diffusion method, shown as diameter of growth inhibition. Color area = size of growth inhibition zone. Data are presented as median values (n = 3), *p* < 0.05.

Among the propylene glycol (PG) extracts, all four preparations corresponding to different harvest months (DEEJune-PG, DEEAug-PG, DEESep-PG, and DEEOct-PG) demonstrated notable activity against Gram-positive microorganisms, particularly exhibiting efficacy against a non-biofilm-forming strain of *Staphylococcus epidermidis*. The incorporation of 1% ascorbic acid into these extracts (DEEJune-PG+Asc, DEEAug-PG+Asc, DEESep-PG+Asc, and DEEOct-PG+Asc) did not result in an enhancement of the inhibition zone diameters. Significantly, extracts derived from plant material harvested in October (DEEOct-PG and DEEOct-PG+Asc) displayed the most potent antimicrobial effects within this series, compared to the extract with the same solvent (*p* < 0.05). These October PG extracts were effective against all tested Gram-positive bacteria and also showed activity against certain clinical Gram-negative isolates, including *Klebsiella pneumoniae* and *Escherichia coli*. However, none of the PG extracts exhibited inhibitory activity against the tested *Candida species*, *Aspergillus niger*, *Pseudomonas aeruginosa*, or *Lactobacillus fermentum* (Figure 6).

A similar profile of antimicrobial action was observed for the glycerin-based (GL) extracts. All GL preparations (DEEJune-GL, DEEAug-GL, DEESep-GL, and DEEOct-GL), including their counterparts supplemented with 1% ascorbic acid (DEEJune-GL+Asc, DEEAug-GL+Asc, DEESep-GL+Asc, and DEEOct-GL+Asc), were active against the tested Gram-positive microorganisms. The most pronounced activity was again noted against the non-biofilm-forming *Staphylococcus epidermidis* strain. Consistent with the PG extracts, the addition of 1% ascorbic acid did not augment the zones of inhibition. The October extracts (DEEOct-GL and DEEOct-GL+Asc) proved to be the most effective among these glycerin-solubilized preparations, demonstrating activity against all tested Gram-positive bacteria and selected clinical Gram-negative organisms (*Klebsiella pneumoniae* and *Escherichia coli*). These GL extracts were also inactive against *Candida species*, *Aspergillus niger*, *Pseudomonas aeruginosa*, and *Lactobacillus fermentum* (Figure 6).

Overall, a positive correlation was evident in the antimicrobial performance of extracts prepared with either glycerin or propylene glycol, with those derived from plant material collected in October consistently demonstrating superior efficacy. The essential oil fraction from *R. tomentosum* exhibited pronounced, broad-spectrum antimicrobial activity, effectively inhibiting the growth of *Aspergillus niger*, as well as both Gram-positive and Gram-negative bacteria, in some instances leading to complete inhibition of visible growth across the inoculated agar surface.

Minimum Inhibitory Concentrations (MICs) were determined for the most promising extracts (DEEOct-PG, DEEOct-PG+Asc, DEEOct-GL, DEEOct-GL+Asc, and DEESept) against selected staphylococcal strains. These lead extracts demonstrated inhibitory activity up to a 1:4 dilution of their initial tested concentration. Several studies have documented the antimicrobial potential of post-distillation residues. For example, wastes derived from *Origanum vulgare*, *Salvia rosmarinus*, *Mentha spicata*, *Salvia fruticosa*, and *Melissa officinalis* exhibit antimicrobial activity and have potential as natural preservatives in food, cosmeceutical, and pharmaceutical applications [62,63,64,65].

**Figure 7:**
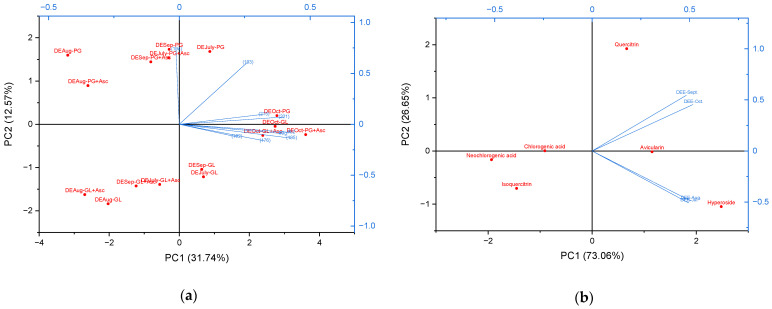
Biplot of the principal component analysis: (**a**) evaluation of the antimicrobial activity of the dry extracts of *Rhododendron tomentosum* Harmaja; (**b**) evaluation of the phenolic content of the dry extracts of *Rhododendron tomentosum* Harmaja.

PCA was employed for a comprehensive evaluation of the antimicrobial activity profiles of *R. tomentosum* dry extracts (Figure 7a). The first two principal components, PC1 and PC2, collectively explained 44.31% of the total data variance (31.74% and 12.57%, respectively). This level of explained variance is characteristic of complex biological datasets where numerous independent variables contribute to the model. Given that this analysis incorporated fourteen distinct microbial strains, the total variance is naturally distributed across several principal components rather than being concentrated solely in the first two [66]. The biplot displays sample scores (red markers, indicating extract distribution) and variable loadings (blue vectors, representing the influence of specific antimicrobial activities). A distinct separation of extracts was observed, primarily along PC1. Extracts derived from *R. tomentosum* material harvested in October (e.g., DEEOct-PG, DEEOct-GL, DEEOct-PG+Asc, DEEOct-GL+Asc) clustered on the positive side of PC1.

This positioning correlated strongly with positive loadings for variables representing antimicrobial efficacy against key Gram-positive bacteria (such as *Staphylococcus aureus* and *Staphylococcus epidermidis*) and certain Gram-negative bacteria (including *Klebsiella pneumoniae* and *Escherichia coli*). This highlights the enhanced activity of October extracts against these particular microorganisms. Conversely, extracts from other collection periods were generally situated on the negative side of PC1, indicating a weaker association with these antimicrobial efficacy markers. Thus, PCA underscored the superior and distinct antimicrobial profile of the October extracts.

The PCA biplot evaluation of the phenolic content of the dry extracts DEE shows a clear separation of phenolic compounds along PC1 and PC2, with flavonol glycosides (hyperoside, avicularin, quercitrin) contributing most strongly to the variability of the extracts (Figure 7b). Hydroxycinnamic acids (chlorogenic and neochlorogenic acids) are positioned on the opposite side of PC1, indicating distinct accumulation patterns. Comparing the general trend of increasing total phenolic and flavonoid content, as well as the levels of individual identified compounds in raw materials harvested in September and October, with the results of microbiological studies, we can conclude that quercetin flavonol glycosides are largely responsible for the antimicrobial effect of the dry extracts.

## 3. Materials and Methods

### 3.1. Chemicals

All the chemicals and reagents used were of analytical grade. Ethanol, ethyl acetate, formic acid, acetonitrile, trifluoroacetic acid, aluminum chloride hexahydrate, Folin–Ciocalteu reagent, and sodium carbonate were purchased from Poch S.A. (Gliwice, Poland). The standards of chlorogenic acid, neochlorogenic acid, hyperoside, isoquercitrin, quercitrin, avicularin were purchased from Sigma-Aldrich (St. Louis, MO, USA).

### 3.2. Plant Material

The aerial parts of *R. tomentosum* (without flowers; without separating into young and last-years shoots) at various vegetative stages were collected from July through October 2023 on the same day of each month in the forest of the Rivne region (Ukraine). The identity of the raw material was established by Nadiia Kovalska, Assoc. Prof. of the Department of Pharmacognosy and Botany of Bogomolets National Medical University, Kyiv. The voucher specimens of the plant have been deposited in the herbarium of the department. The shoots were shade-dried at room temperature and stored in tightly closed containers. The samples (a mixture of stems and leaves) were crushed before the essential oils were isolated (particle size 5–10 mm).

### 3.3. Sample Preparation

Hydrodistillation was carried out with a circulatory Clevenger-type apparatus according to the European Pharmacopoeia (without adding xylene). In one apparatus, approximately 50 g of air-dried crushed raw materials were dipped in 1000 mL of distilled water, so the ratio of shoots to water was 1:20. Hydrodistillation was performed for 2 h. A mixture of essential oils and water was collected in 5 mL of dichloromethane, which was then separated into water and dichloromethane phase by a separatory funnel. The water layer was extracted with 5 mL dichloromethane twice, and all three fractions were combined. The dichloromethane phase containing volatile compounds was dried over anhydrous magnesium sulfate and concentrated using a rotary evaporator at 35 °C to a final volume of 1.0 mL. An aliquot of 1 µL was then analysed by GC-MS.

Post-distillation residues obtained after hydrodistillation, including both liquid and solid waste, were analysed for residual amounts of biologically active substances. For this, the aqueous extract was evaporated to a dry extract (named DEA), and condensed water was also obtained in this way (named liquid fraction after evaporation). The solid waste of plant materials was macerated with 80% ethanol for 24 h, followed by sonication for 30 min. The resulting ethanolic extract was then evaporated to obtain a dry extract (named DEE).

Thus, four types of products were obtained by hydrodistillation: essential oil, liquid fraction after evaporation of wastewater, and two dry extracts (DEA and DEE), prepared from plant raw materials collected in July, August, September, and October.

For GC-MS analysis of the liquid fraction obtained after evaporation wastewater, the following sample preparation was performed. The liquid fraction was transferred into a separation funnel and extracted with three portions of dichloromethane. The dichloromethane phases were combined and filtered through layers of anhydrous magnesium sulfate. Then, they were concentrated on a rotary evaporator at 35 °C to a final volume of 1 mL, and 1 µL was analysed using GC-MS.

HPLC analysis of the dry extracts DEA and DEE, approximately 0.05 g of extract was dissolved in 2,5 mL of water or ethanol 80%, respectively. For chromatographic studies, the extract solution was additionally filtered through a Millipore filter with a pore size of 0.45 µm. 10 µL of the obtained solution was analysed using HPLC-PDA.

For spectrophotometricanalysis of the dry extracts DEA and DEE, approximately 0.02 g of extract was dissolved in 5 mL of water or ethanol 80%, respectively. These stock solutions of extract were used to study the phenolic and flavonoid total content and DPPH test.

For the microbiological studies, only the DEE extracts obtained from solid waste were used. Approximately, 0.01 g of the extract was dissolved in 2.0 g of propylene glycol (PG) and glycerin (GL) separately (samples named DEEJuly-PG, DEEAug-PG, DEESep-PG, DEEOct-PG, DEEJuly-GL, DEEAug-GL, DEESep-GL, and DEEOct-GL). Ascorbic acid was added to parallel solutions at a concentration of 1% (samples named DEEJuly-PG+Asc, DEEAug-PG+Asc, DEESep-PG+Asc, DEEOct-PG+Asc, DEEJuly-GL+Asc, DEEAug-GL+Asc, DEESep-GL+Asc, and DEEOct-GL+Asc). Essential oil and liquid fraction after evaporation were also tested. The general research plan is presented in Figure 1.

### 3.4. GC-MS Analysis

GC–MS analyses were performed as previously described [67,68] with slight modifications. The essential oils and liquid waste were analysed using a gas chromatograph HP 6890 system (Hewlett-Packard, Böblingen, Germany) coupled to a 5973 mass selective detector (Hewlett-Packard, Böblingen, Germany). Separation was achieved on a ZB-5HT capillary column ((5–phenyl)-methylpolysiloxane, 60 m × 0.25 mm i.d., 0.25 µm film thickness; Phenomenex Inc., Torrance, CA, USA). The injector temperature was set at 250 °C. The mass spectrometer operated in electron impact ionization mode at 70 eV, with a scan range of 30–450 m/z and an ion source temperature of 230 °C. Helium was used as the carrier gas at a constant flow rate of 2.0 mL/min. The oven temperature program was as follows: initial temperature of 50 °C held for 5 min, increased to 250 °C at 3 °C/min, then to 280 °C at 10 °C/min.

Compound identification was based on the comparison of mass spectra with those in the NIST 11 and NIST 14 library (National Institute of Standards and Technology, Gaithersburg, MD, USA), using match probabilities ≥80%. In addition, linear retention indices (RI) calculated according to Van den Dool and Kratz formula relative to a mixture of n-alkanes (C_8_–C_29_) eluted on the same column using the Excel tool “Retentify” [69] and compared with reference values from the literature [35,36]. Each sample was analysed in triplicate, and results are presented as average peak areas.

### 3.5. Determination of Total Phenolic Content

Total phenolic content (TPC) was determined by using the Folin–Ciocalteu method as described previously [24] with slight modifications. 0.1 mL of the stock solution (diluted with distilled water to a suitable concentration) was placed in a 2 mL Eppendorf tube, and 0.1 mL of 1:1 diluted in water Folin–Ciocalteu reagent was added. Then, 0.2 mL of saturated Na_2_CO_3_ solution (106.0 g/L) and 1.6 mL of distilled water were added. After incubation at room temperature for 120 min, the test samples were subjected to spectral analysis in the range 550–850 nm against the blank using Rayleigh UV–vis spectrophotometer (Beijing Beifen-Ruili Analytical Instrument Co., Ltd., Beijing, China). The blank consisted of the same volume of the tested sample, sodium carbonate solution, and 1.7 mL of distilled water. All analyses were performed in triplicate.

Gallic acid was used as the standard for a calibration curve, and the results were expressed as mg of gallic acid equivalents per g of dry extract (mg GAE/g dry extract). Data were expressed as mean ± standard deviation (SD%). TPC was calculated by the standard method in terms of gallic acid according to the formula:

$$X,\;mg/g=\frac{A\cdot m_{st}\cdot K}{A_{st}\cdot m}\cdot 1000$$

where X is the total phenolic content in terms of gallic acid, mg/g;

K—dilution factor;

A—absorption of diluted sample at 760 nm;

A_st_—absorption of solutions of gallic acid at 760 nm;

m—mass of the extract sample;

m_st_—mass of the standard substance.

Calibration range of gallic acid was from 1.45 to 10.08 µg/mL. The calibration curve was made by linear regression R2 = 0.9959 (y = 9.9081x + 0.4594), LOD 0.9 µg/mL, LOQ 2.7 µg/mL.

### 3.6. Determination of Total Flavonoid Content

The total flavonoid content (TFC) was determined using the colorimetric method described previously [24], with some modifications. 1 mL of the stock solution (diluted with distilled water to a suitable concentration) was mixed with 1 mL of 10% AlCl_3_ water solution in a 2 mL Eppendorf tube. The mixture was vortexed well for 10 s and incubated for 60 min at room temperature in a dark place. The absorbance was measured in the range 350–500 nm relative to a blank. The blank consisted of the same volume of the tested sample and 1 mL of distilled water. The results were expressed as mg of rutin and quercetin equivalents per g of dry extract (mg RE/g, mg QE/g). The total flavonoid content was calculated using the formula described above, where A and Ast were absorption at 410 nm in terms of rutin and at 425 nm for calculation in terms of quercetin. All analyses were performed in triplicate and data were expressed as mean ± standard deviation (SD%). The calibration range of rutin was from 7.6 to 60.8 µg/mL, the calibration curve was made by linear regression R2 = 0.9955 (y = 64.147x − 5.0179), LOD 0.9 µg/mL, LOQ, 2.9 µg/mL. The calibration range of quercetin was from 1.1 to 11.3 µg/mL (R2 = 0.9929, y = 16.969x + 0.6726), LOD 0.6 µg/mL, LOQ 2.0 µg/mL.

### 3.7. HPLC–PDA Analysis

HPLC analysis was performed using a Waters e2695 chromatograph (Waters Corporation, Milford, CT, USA) equipped with a Waters 2998 PDA photodiode array detector (Waters Corporation). Separation of the polyphenolic compounds was achieved on an ACE C18 (250 × 4.6 mm, 5 µm; Advanced Chromatography Technologies, Aberdeen, UK). The column was temperature-controlled, with a temperature maintained at 25 °C. The injection volume was 10 µL, and the elution flow rate was 1.0 mL/min. A gradient elution was carried out using eluent A—0.1% (*v*/*v*) trifluoroacetic acid (TFA), and eluent B—100% acetonitrile. The following linear gradient program was applied: 0 min, 95% A and 5% B; 8 min, 85% A and 15% B; 30 min, 80% A and 20% B; 48 min, 60% A and 40% B; 58 min, 50% A and 50% B; 66 min, 5% A and 95% B; 70 min, 5% A and 95% B; and 71 min, 95% A and 5% B. The identification of chromatographic peaks was performed by comparing the retention times and UV absorption spectra (210–400 nm) of the analytes with those of reference standard compounds. The detection of phenolic acids and flavonoids was carried out at wavelengths of 325 and 355 nm, respectively.

Standard stock solutions were prepared in a 70% methanol−water mixture (*v*/*v*) at concentrations of 0.1 mg/g for hyperoside, isoquercitrin, quercitrin, and avicularin, and 0.2 mg/g chlorogenic acid and neochlorogenic acid. These solutions were subsequently diluted to six different concentrations. The contents of phenolic acids were calculated at a wavelength of 325 nm, and the contents of flavonoids at 355 nm. The results of the phytochemical analysis are expressed as micrograms per gram of dry weight (µg/g DW). Validation of the HPLC–PDA method was performed according to the following parameters: specificity, linearity, precision, LOD, and LOQ (Table 5).

### 3.8. Radical Scavenging Activity (DPPH Test)

The antiradical activity of dry extracts DEA and DEE was evaluated using the method described previously [24], with some modifications. 0.05 mL of the stock solution (diluted with ethanol 96% to a suitable concentration) was added to 1.95 mL of an ethanolic solution of DPPH (0.1 mM) in a 2 mL Eppendorf tube. The mixture was incubated for 30 min at room temperature in a dark place, and the absorbance was measured in the range 400–600 nm against a blank. The blank consisted of the same volume of the tested sample and 1.95 mL of 96% ethanol. A control experiment was run with DPPH solution without the tested sample against ethanol. The antiradical activity (%) of the dry extracts was calculated using the following formula:

$$DPPH\;radical-scavenging\;activity,\left(\%\right)=\frac{A_{control}-A_{sample}}{A_{control}}\cdot 100$$

where Abs is absorbance at 517 nm.

The radical-scavenging activity percentage of the test samples was compared to that of the gallic acid and quercetin standards, and the results were expressed in terms of gallic acid equivalent antioxidant capacity (GEAC) and quercetin equivalent antioxidant capacity (QEAC) in mg gallic acid and mg quercetin equivalents per g of dry extract. All determinations were carried out three times and data were expressed as mean ±standard deviation (SD%). The calibration range of gallic acid was from 1.4 to 10.1 µg/mL, and the calibration curve was made by linear regression R2 = 0.9945 (y = 9.9081x − 0.4594), LOD 0.9 µg/mL, LOQ 2.7 µg/mL. The calibration range of quercetin was from 0.6 to 3.4 µg/mL (R2 = 0.9924, y = 17.65x − 0.0557), LOD 0.17 µg/mL, LOQ 0.52 µg/mL.

### 3.9. Antimicrobial Activity In Vitro

The in vitro antibacterial and antifungal activities of the investigated extracts were evaluated using the agar disc diffusion method, following established guidelines [70,71]. Briefly, 25 µL of each extract solution were impregnated onto sterile paper discs (5.5 mm diameter; SD067 Sterile discs, Himedia Laboratories Pvt. Limited, Maharashtra, India). These discs were then carefully placed on the surface of agar plates previously seeded with a microbial suspension (0.5 McFarland). The pure solvent used to dissolve the extracts served as a negative control. Mueller-Hinton Agar (MHA) (Sigma-Aldrich, St. Louis, MO, USA) was utilized for bacterial cultures, Sabouraud Dextrose Agar (SDA) (Sigma-Aldrich, St. Louis, MO, USA) for fungal cultures, and De Man, Rogosa and Sharpe (MRS) Agar (HiMedia Laboratories, LLC, Thane, India) for *Lactobacillus* strain. The inoculated plates were incubated at 37 °C for 24 h for bacteria, and at 25 °C for 24–48 h for fungi. Antimicrobial activity was assessed by measuring the diameter (in mm) of the clear inhibition zone formed around each disc.

The Minimum Inhibitory Concentration (MIC) values of the extracts were determined using a resazurin-based microdilution assay in 96-well microtiter plates, adapted from previously described methods [71,72]. In each well, 50 µL of the appropriate liquid growth medium (Mueller-Hinton Broth for bacteria or glucose-supplemented Nutrient Broth for yeasts/molds), 50 µL of the microbial suspension (0.5 McFarland), and 100 µL of the test extract (at various concentrations, typically prepared by serial dilution) were combined. The plates were then incubated under optimal conditions (e.g., 37 °C for 24 h for bacteria; 25 °C for 24–48 h for fungi). Following this incubation, 15 µL of a 0.02% (*w*/*v*) resazurin solution were added to each well, and the plates were incubated for an additional 2–4 h to allow for color development. The MIC was defined as the lowest concentration of the extract that prevented the metabolic reduction in resazurin (i.e., no color change from blue to pink), indicating inhibition of microbial growth.

A panel of fourteen microbial strains, encompassing both reference and clinical isolates, was employed in this study. This collection included five Gram-positive bacteria, five Gram-negative bacteria, two Candida species, one mold fungus (*Aspergillus niger*), and one probiotic bacterial strain. All strains were identified using MALDI-TOF mass spectrometry (Bruker, Bremen, Germany) or 16S rRNA gene sequences. All clinical isolates were characterized as multidrug-resistant (MDR) or extensively drug-resistant (XDR), exhibiting diverse antimicrobial resistance (AMR) profiles. Notably, two biofilm-forming *Staphylococcus* strains were found to harbor the pox gene, which is associated with biofilm formation capabilities. Strain b2 was identified as methicillin-resistant *Staphylococcus aureus* (MRSA), and *Klebsiella pneumoniae* strain 215 was confirmed as a producer of both Extended-Spectrum β-Lactamases (ESBL) and *Klebsiella pneumoniae* Carbapenemase (KPC). All antimicrobial susceptibility tests were performed in triplicate.

### 3.10. Statistical Analysis

Statistical analysis was performed using the MS Office Excel v. 2010, while charts were produced in OriginPro 2025 environment. The mean and standard deviation (±SD) were calculated according to the monograph “Statistical Analysis of the Results of a Chemical Experiment” of the State Pharmacopoeia of Ukraine) [73]. The average value was established based on 3 measurements.

## 4. Conclusions

This study demonstrates that both the essential oil and post-distillation by-products of *R. tomentosum* are valuable sources of bioactive compounds with antioxidant and antimicrobial properties. The essential oil, rich in monoterpenes and sesquiterpenes such as terpineol <γ->, palustrol, ledol, and cymene <ρ->, showed considerable chemical variability depending on the month of plant collection. In parallel, the by-products obtained from hydrodistillation, dry extracts obtained from solid (DEE) and liquid (DEA) residues, were found to contain significant amounts of polyphenols and flavonoids. DEE extracts exhibited higher TPC and TFC values as well as stronger antioxidant capacity compared to DEA. HPLC analysis confirmed the presence of key polyphenolic substances, including neochlorogenic acid, chlorogenic acid, hyperoside, isoquercitrin, quercitrin, and avicularin. Furthermore, antimicrobial assessment and principal component analysis revealed that several of the extracts, particularly those prepared from October harvested material and formulated with glycerin and propylene glycol, possessed enhanced antimicrobial activity. The essential oil of *R. tomentosum* exhibited significant efficacy against *Aspergillus niger*, highlighting its antifungal potential. In contrast, the liquid fraction obtained after evaporation of wastewater was mostly inactive. These highlight the importance of valorising post-distillation plant residues as functional ingredients with potential applications in food preservation, pharmaceuticals, and natural antimicrobial formulations.

Analyses performed after one year of storage confirmed the overall stability of the dry extracts obtained from post-distillation residues of *R. tomentosum*. Although a partial decline in total polyphenol and flavonoid contents (approximately 5–20%) and a noticeable reduction in antioxidant capacity were observed. Despite some loss of activity, the extracts retained considerable levels of bioactive compounds and antioxidant potential after prolonged storage. These results emphasize the value of post-distillation by-products as stable sources of natural antioxidants and antimicrobial agents for applications in food, nutraceutical, and pharmaceutical formulations, although further studies are required.

## Figures and Tables

**Table 1:** Essential oil and by-product yields obtained from shoots of *Rhododendron tomentosum* Harmaja.

Extracts	Yield, %			
	July	August	September	October
Essential oil	0.8	0.8	1.0	0.9
Liquid fraction obtained after evaporation of the wastewater	92	94	90	89
Dry extract DEA	17.4	18.3	15.6	17.1
Dry extract DEE	4.7	5.5	5.6	4.4

**Table 2:** GC-MS profile of the essential oils of *Rhododendron tomentosum* Harmaja.

^a^ Compound	^b^ RI_Exp._	^c^ RI_Lit._	^d^ RI_Lit._	Peak Area (%)			
				July	August	September	October
Hexanal	799	801	800	-	-	-	0.1 ± 0.1
Hexenal <(2E)->	850	846	853	0.2 ± 0.2	0.1 ± 0.2	-	0.2 ± 0.1
Hexenol <(3E)->	853	844	853	-	0.2 ± 0.1	-	0.1 ± 0.1
Pinene <α->	937	932	936	0.3 ± 0.1	0.4 ± 0.1	0.4 ± 0.2	0.3 ± 0.1
Camphene	953	946	950	-	0.3 ± 0.1	0.7 ± 0.2	0.2 ± 0.1
Sabinene	977	969	973	-	0.7 ± 0.2	-	0.3 ± 0.1
Pinene <β->	982	974	978	0.4 ± 0.1	0.6 ± 0.2	0.6 ± 0.1	0.4 ± 0.1
Myrcene	991	988	989	-	0.3 ± 0.1	-	-
Phellandrene <α->	1008	1002	1004	-	0.1 ± 0.1	-	-
Terpinene <α->	1021	1014	1017	1.3 ± 0.2	4.2 ± 0.2	4.1 ± 0.2	1.3 ± 0.1
Cymene <ρ->	1030	1020	1024	9.9 ± 0.4	9.7 ± 0.3	9.0 ± 0.2	9.4 ± 0.2
Limonene	1033	1024	1030	0.6 ± 0.1	0.9 ± 0.1	0.9 ± 0.1	0.6 ± 0.1
Phellandrene <β->	1035	1025	1030	0.3 ± 0.1	0.6 ± 0.2	0.5 ± 0.2	0.4 ± 0.1
Ocimene <(Z)-β->	1037	1032	1038	0.4 ± 0.1	0.7 ± 0.2	0.5 ± 0.1	0.4 ± 0.1
Ocimene <(E)-β->	1048	1044	1048	-	0.6 ± 0.2	0.5 ± 0.1	0.3 ± 0.1
Terpinene <γ->	1062	1054	1060	1.2 ± 0.3	2.3 ± 0.2	2.3 ± 0.3	1.2 ± 0.2
Terpinolene	1093	1086	1087	0.6 ± 0.1	0.8 ± 0.2	0.7 ± 0.2	0.3 ± 0.1
Cymenene <ρ->	1094	1089	1088	-	-	-	0.4 ± 0.1
Linalool	1101	1095	1099	1.7 ± 0.2	0.9 ± 0.2	0.9 ± 0.2	1.5 ± 0.2
Nonanal <n->	1104	1100	1103	0.3 ± 0.1	0.1 ± 0.1	-	0.3 ± 0.1
Menth-2-en-1-ol <cis-ρ->	1127	1118	1123	0.5 ± 0.1	0.4 ± 0.1	0.3 ± 0.01	0.3 ± 0.2
Campholenal <α->	1132	1122	1124	-	0.2 ± 0.1	-	-
p-Mentha-1,5,8-triene	1142	1133	1131	-	0.3 ± 0.1	-	0.3 ± 0.1
Menth-2-en-1-ol <trans-ρ->	1145	1136	1137	-	0.2 ± 0.1	-	0.2 ± 0.1
Pinocarveol <trans->	1148	1135	1140	0.8 ± 0.2	0.6 ± 0.1	0.6 ± 0.1	0.9 ± 0.2
Pinocarvone	1171	-	1161	0.7 ± 0.1	0.6 ± 0.2	0.6 ± 0.1	0.6 ± 0.2
Borneol	1175	1165	1166	0.5 ± 0.1	0.4 ± 0.1	0.4 ± 0.1	0.9 ± 0.2
Terpinen-4-ol	1185	1174	1177	2.7 ± 0.3	2.6 ± 0.4	2.8 ± 0.3	2.2 ± 0.3
Thuj-3-en-10-al	1191	1181	-	-	0.3 ± 0.1	-	-
Terpineol <α->	1198	1186	1190	0.4 ± 0.1	0.3 ± 0.1	-	0.3 ± 0.1
Piperitol <cis->	1203	1195	1196	-	0.2 ± 0.1	-	-
Myrtenal	1205	1195	1192	1.3 ± 0.2	0.8 ± 0.2	0.8 ± 0.1	1.7 ± 0.2
Terpineol <γ->	1250	1199	-	25.3 ± 1.2	34.5 ± 2.5	36.9 ± 2.3	21.0 ± 1.8
Geraniol	1257	1249	1255	0.6 ± 0.2	1.2 ± 0.2	1.2 ± 0.3	1.6 ± 0.4
Bornyl acetate	1294	1286	1284	4.4 ± 0.2	4.4 ± 0.2	4.3 ± 0.2	4.7 ± 0.3
Isoascaridol	1316	-	-	0.9 ± 0.1	1.3 ± 0.3	1.2 ± 0.2	0.8 ± 0.1
Not identified	1353	-	-	1.7 ± 0.3	1.4 ± 0.2	1.4 ± 0.2	1.8 ± 0.2
Geranyl acetate	1385	1379	1380	5.8 ± 0.2	5.4 ± 0.2	5.6 ± 0.2	5.6 ± 0.2
Bourbonene <β->	1398	1387	1384	-	-	-	0.2 ± 0.1
Gurjunene <α->	1424	1409	1409	1.4 ± 0.3	0.9 ± 0.2	0.8 ± 0.1	1.5 ± 0.3
Caryophyllene <(E)->	1435	1417	1420	0.5 ± 0.1	-	-	0.5 ± 0.1
Gurjunene <β->	1449	1431	1431	-	0.2 ± 0.1	-	0.3 ± 0.1
Farnesene <(Z)-β->	1459	1440	1446	-	-	-	0.3 ± 0.1
Aromadendrene <allo->	1477	1458	1460	5.9 ± 1.3	2.8 ± 0.5	2.8 ± 0.5	5.9 ± 1.1
Germacrene D	1496	1480	1481	-	0.3 ± 0.2	-	0.6 ± 0.1
Viridiflorene	1502	1496	1492	0.7 ± 0.1	-	-	0.8 ± 0.1
Shyobunone	1526	-	-	-	-	-	0.2 ± 0.1
Cadinene <δ->	1535	1522	1523	-	-	-	0.4 ± 0.1
Palustrol	1588	1567	1567	17.2 ± 1.2	10.8 ± 2.3	12.2 ± 2.8	16.1 ± 3.2
Ledol	1604	1602	1567	8.1 ± 1.8	4.1 ± 1.6	3.6 ± 1.4	8.0 ± 2.1
Monoterpene hydrocarbons				14.9	22.5	20.2	15.2
Oxygenated monoterpenes				46.8	43.3	56.6	44.2
Sesquiterpene hydrocarbons				8.5	4.2	3.6	9.9
Oxygenated sesquiterpenes				25.3	12.9	12.7	24.6
Other				0.23	0.3	-	0.4
Not identifier				4.27	16.8	6.9	5.7

^a^ Order of compounds is according to elution from ZB-5HT column. ^b^ Linear retention indices calculated according to Van den Dool and Kratz formula relative to a mixture of n-alkanes (C_8_–C_29_) eluted on apolar ZB-5HT column. ^c^ Linear retention index for an apolar column taken from Adams (2007) [35]. ^d^ Linear Retention index for an apolar column taken from Babushok (2011) [36].

**Table 3:** GC-MS profile of the liquid fraction obtained after evaporation of the wastewater.

^a^ Compound	^b^ RI_Exp_.	^c^ RI_Lit._	^d^ RI_Lit._	Peak Area (%)			
				July	August	September	October
Hexenol <(3Z)->	860	850	857	-	-	0.6 ± 0.1	-
Benzyl alcohol	1037	1026	1037	-	1.2 ± 0.2	0.7 ± 0.2	1.0 ± 0.2
Cresol <ρ->	1076	1071	1077	2.1 ± 0.3	2.1 ± 0.2	1.7 ± 0.3	1.9 ± 0.3
Linalool	1101	1095	1099	2.3 ± 0.3	0.9 ± 0.2	1.5 ± 0.4	1.5 ± 0.4
Phenyl ethyl alcohol	1119	1106	1115	3.1 ± 0.4	5.9 ± 0.2	3.5 ± 0.4	3.5 ± 0.3
Sabina ketone <dehydro->	1127	1117	-	2.4 ± 0.2	1.9 ± 0.3	0.4 ± 0.2	2.6 ± 0.3
Pinocarveol <trans->	1148	1135	1140	-	1.1 ± 0.2	1.3 ± 0.2	-
Pinene oxide <β->	1165	1154	1156	-	0.5 ± 0.1	0.5 ± 0.1	-
Borneol	1175	1165	1166	-	0.7 ± 0.2	1.4 ± 0.3	-
Terpinen-4-ol	1185	1174	1177	5.7 ± 1.2	1.9 ± 0.8	3.2 ± 1.4	3.1 ± 1.6
Cymen-8-ol <ρ->	1191	1179	1184	4.5 ± 1.3	3.3 ± 1.1	3.3 ± 1.1	3.5 ± 1.1
Cryptone	1195	1183	1184	-	-	0.5 ± 0.1	-
Terpineol <α->	1197	1186	1190	-	1.1 ± 0.2	1.2 ± 0.2	-
Myrtenal	1204	1195	1194	-	-	1.2 ± 0.2	-
Ascaridole	1248	1234	-	4.1 ± 1.2	0.5 ± 0.3	1.4 ± 0.8	-
Geraniol	1256	1249	1255	2.5 ± 1.0	2.2 ± 1.2	2.9 ± 1.3	2.9 ± 1.2
Piperitone epoxide <trans->	1263	1252	-	-	0.7 ± 0.2	0.8 ± 0.2	-
Cymen-7-ol <ρ->	1298	1289	1288	-	0.8 ± 0.2	0.7 ± 0.2	-
Fenchol <2-ethyl-exo->	1303	1297	-	-	1.2 ± 0.3	-	-
Thymol	1308	1289	1290	2.4 ± 0.6	1.4 ± 0.8	0.7 ± 0.3	2.9 ± 1.1
2,4,4,7-Tetramethyl-5,7-octadien-3-ol	1312	-	-	25.7 ± 2.3	23.5 ± 3.5	25.1 ± 3.1	26.3 ± 2.8
Geranyl acetate	1383	1379	1380	-	-	0.5 ± 0.1	-
Palustrol	1587	1567	1567	9.2 ± 1.3	5.7 ± 0.8	3.7 ± 1.0	5.8 ± 1.8
Ledol	1624	1602	1567	7.4 ± 2.1	4.9 ± 1.3	3.1 ± 0.8	4.8 ± 1.2
Cyperotundone	1781	1695	-	2.9 ± 1.1	2.6 ± 1.0	1.5 ± 0.8	2.4 ± 1.1
Oxygenated monoterpenes				23.8	17.0	20.8	16.6
Oxygenated sesquiterpenes				16.6	10.6	7.3	10.5
Other				5.2	10.4	6.5	6.4
Not identifier				54.4	62.1	65.4	66.5

^a^ Order of compounds is according to elution from ZB-5HT column. ^b^ Linear retention indices calculated according to Van den Dool and Kratz formula relative to a mixture of n-alkanes (C_8_–C_29_) eluted on apolar ZB-5HT column. ^c^ Linear retention index for an apolar column taken from Adams (2007) [35]. ^d^ Linear Retention index for an apolar column taken from Babushok (2011) [36].

**Table 4:** Content of phenolic compounds in the dry extracts DEA and DEE of *Rhododendron tomentosum* Harmaja determined by HPLC-PDA methods.

Peak No.	Compounds	Dry Extracts, mg/g DW ± SD							
		July		August		September		October	
		DEA	DEE	DEA	DEE	DEA	DEE	DEA	DEE
1	Neochlorogenic acid	1.53 ± 0.15	1.01 ± 0.09	0.48 ± 0.04	0.57 ± 0.06	1.22 ± 0.12	0.99 ± 0.11	2.67 ± 0.36	1.86 ± 0.17
2	Chlorogenic acid	3.32 ± 0.33	2.40 ± 0.21	1.15 ± 0.11	1.53 ± 0.12	2.90 ± 0.29	2.69 ± 0.31	3.80 ± 0.51	3.12 ± 0.30
3	Hyperoside	1.62 ± 0.16	7.89 ± 0.71	1.07 ± 0.10	8.37 ± 0.96	0.98 ± 0.09	5.40 ± 0.63	1.61 ± 0.21	6.16 ± 0.59
4	Isoquercitrin	0.32 ± 0.03	2.13 ± 0.19	0.19 ± 0.01	2.33 ± 0.26	0.23 ± 0.02	0.95 ± 0.11	0.34 ± 0.04	1.75 ± 0.16
5	Avicularin	0.76 ± 0.07	1.56 ± 0.14	0.29 ± 0.02	1.48 ± 0.17	1.49 ± 0.14	7.33 ± 0.86	2.19 ± 0.29	7.12 ± 0.68
6	Quercitrin	0.69 ± 0.06	4.57 ± 0.41	0.48 ± 0.4	5.19 ± 0.59	0.55 ± 0.05	5.11 ± 0.60	1.24 ± 016	5.72 ± 0.55

The values are presented as mean ± standard deviation from triplicate measurements, *p* < 0.05.

**Table 5:** Validation data of HPLC-PDA analysis.

Compounds	Range, µg/mL	Formula	LOD, µg/mL	LOQ, µg/mL	RSD RT/Area, %	RSD RT/Area, %	R^2^
Neochlorogenic acid	25–0.195	Y = 55,000x − 2290	0.049	0.102	0.1/0.14	0.2/0.31	0.99982
Chlorogenic acid	208–0.406	Y = 31,000x − 7620	0.051	0.113	0.1/0.15	0.18/0.25	0.99981
Hyperoside	193.8–0.378	Y = 21,000x − 5170	0.189	0.252	0.1/0.25	0.2/0.29	0.99985
Isoquercitrin	187.2–0.731	Y = 21,800x + 7890	0.183	0.283	0.1/0.3	0.21/0.32	0.99977
Quercitrin	205.5–0.401	Y = 19,700x − 10100	0.191	0.201	0.2/0.28	0.25/0.4	0.99982
Avicularin	430–1.679	Y = 23,300x − 5880	0.105	0.212	0.21/0.3	0.3/0.42	0.99998

## Data Availability

The data supporting the reported results can be found in the text and Supplementary Materials.

## Supplementary Materials

The following supporting information can be downloaded at: https://www.mdpi.com/article/10.3390/molecules31101579/s1, Figure S1: (a): HPTLC fingerprints of the dry extracts DEA and DEE of *Rhododendron tomentosum* Harmaja. (b): HPTLC fingerprints after one year of storage.
