# Supplementary material for: Valorization of Aromatic Plant Distillation Residues: Phenolic Composition, Antioxidant Capacity, and Antimicrobial Activity of Rhododendron tomentosum Harmaja Extracts
Source: Molecules. 2026 May 9;31(10):1579. doi: 10.3390/molecules31101579 (PMC13209619; doi:10.3390/molecules31101579)
Supplement: Supplementary file 1 [file molecules-31-01579-s001.zip › molecules-4269704-supplementary.pdf]

Supporting Information

Valorization of Aromatic Plant Distillation Residues: Phenolic Composition, Antioxidant Capacity, and Antimicrobial Activity of *Rhododendron tomentosum* Harmaja Extracts

Izabela Jasicka-Misiak, Halyna Kukhtenko, Yulian Konechnyi, Liudas Ivanauskas, Mindaugas Marksa, Ján Brindza and Oleksandr Kukhtenko

Sample preparation

HPTLC analysis of the dry extracts DEA and DEE, approximately 0.02 g of extract was dissolved in 1 mL of water or ethanol 80%, respectively. For chromatographic studies, the extract solution was additionally filtered through a Millipore filter with a pore size of 0.45 µm. 5 µL of the obtained solution was analysed using HPTLC.

HPTLC Analysis

Test samples (5 µL) and standard solutions (1.5 µL, 1 mg/1.5 mL) were applied to the plates using an automatic HPTLC application device (Linomat 5, CAMAG, Muttenz, Switzerland). Chromatographic separations were performed on 20 cm × 10 cm HPTLC plates (Silica gel 60, 0.25 thickness, Merck) in a vertical glass chamber (CAMAG). Mobile phase: ethyl acetate - formic acid - water (15:1:1). 50 mL of the mobile phase was used, the chamber saturation time with the mobile phase was 20 minutes. After the eluent covered the distance from the start line to the finish line, the plates were removed from the chamber and dried at (105±2)°C. Detection was based on natural fluorescence before and after derivatization by sequentially spraying with 2-aminoethyl diphenylborate (10 g/L) and macrogol 400 (50 g/L) in UV light at 366 nm. The obtained chromatographic images were analysed using HPTLC software (visionCATS, CAMAG).

Polyphenolic profiles of solid and liquid wastes from hydrodistillation

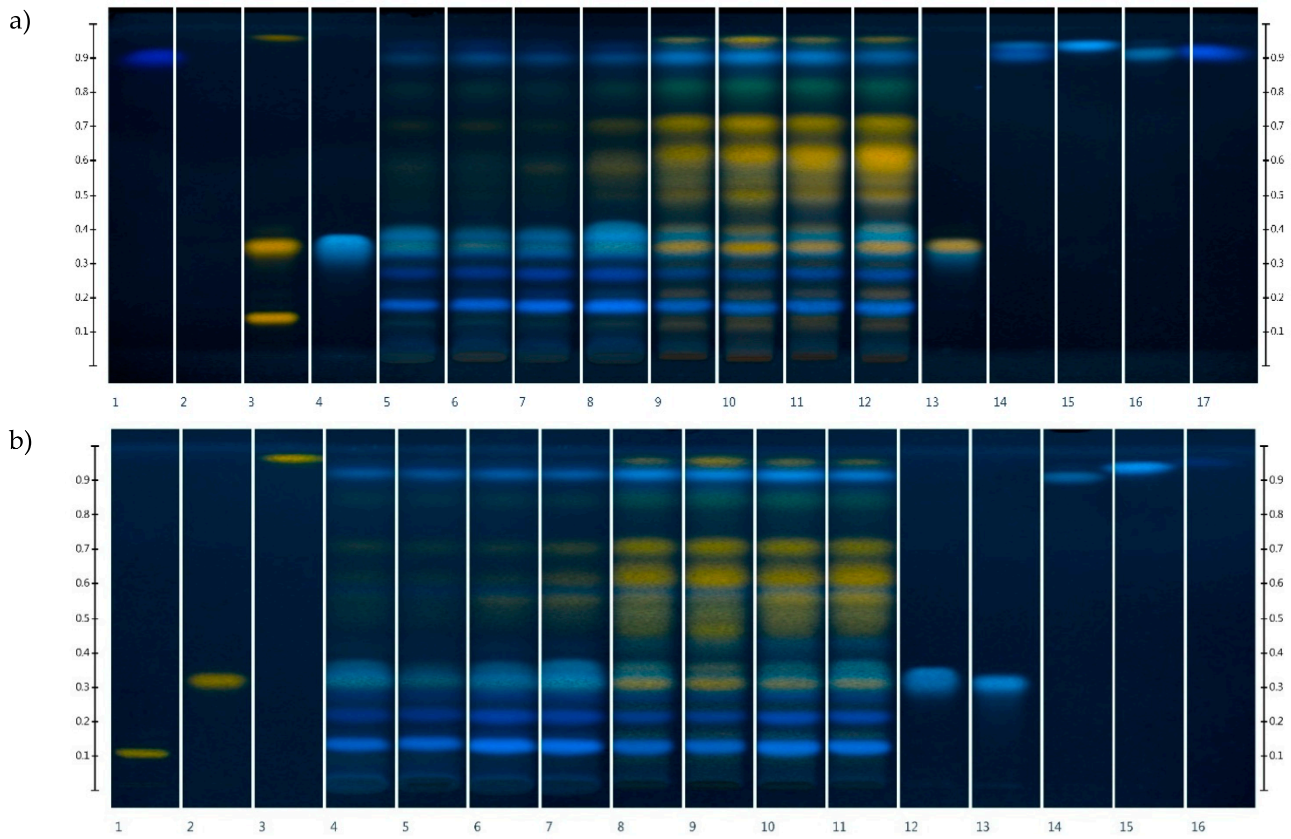

Figure S1. a) HPTLC fingerprints of the dry extracts DEA and DEE of *Rhododendron tomentosum* Harmaja.

Track 1 – Gallic acid, track 2 – Arbutin, track 3 – Rutin + Hyperoside + Quercetin, track 4 – Chlorogenic acid, track 5 – DEA-July, track 6 – DEA-August, track 7 – DEA-September, track 8 – DEA-October, track 9 – DEE-July, track 10 – DEE-August, track 11 – DEE-September, track 12 – DEE-October, track 13 –

Arbutin + Chlorogenic acid + Hyperoside, track 14 – Rosmarinic acid + Scopoletin, track 15 – Caffeic acid, track 16 – Rosmarinic acid, track 17 – Scopoletin.

b) HPTLC fingerprints after one year of storage. Track 1 – Rutin, track 2 – Hyperoside, track 3 – Quercetin, track 4 – DEA-July, track 5 – DEA-August, track 6 – DEA-September, track 7 – DEA-October, track 8 – DEE-July, track 9 – DEE-August, track 10 – DEE-September, track 11 – DEE-October, track 12 – Chlorogenic acid, , track 13 –Neochlorogenic acid, track 14 – Rosmarinic acid, track 15 – Caffeic acid, track 16 – Ferulic acid.

HPTLC was additionally applied to characterize the chemical profiles of the dry extracts. Visual evaluation of the HPTLC plate enabled side-by-side comparison of multiple samples, allowing for the observation of similarities and differences in the sequence, color, and intensity of the separated zones between sample and reference tracks. Figure a) and b) reveal differences in the fingerprint chromatograms of the DEA and DEE dry extracts obtained after the hydrodistillation of *R. tomentosum* shoots. After scanning the chromatographic plate under UV light at 366 nm after derivatization with 2-aminoethyl diphenylborate and detection at 366 nm, zones corresponding in color and retention factor (Rf) to standard compounds chlorogenic acid (Rf 0.37), hyperoside (Rf 0.35) were observed. Chlorogenic acid was present in all extract samples, while hyperoside were detected only in the DEE dry extracts. Chlorogenic acid is the main phenolic acid used to identify *R. tomentosum* plant material according to the German Homoeopathic Pharmacopoeia. Hyperoside and quercetin are recognized as marker compounds in the French Pharmacopoeia. Although the colors of gallic acid, rosmarinic acid, scopoletin, and caffeic acid correspond to some zones in the extract tracks, there is no scientific data to confirm their presence. Comparison of HPTLC fingerprints recorded immediately after extract preparation and after one year of storage revealed no differences in the overall profile. Moreover, comparison with previously published HPTLC fingerprint data of *R. tomentosum* liquid extracts revealed no significant differences in the overall phytochemical profile, indicating thermal stability of the compounds during hydrodistillation [1].

References

1. Kukhtenko, H.; Bevz, N.; Konechnyi, Y.; Kukhtenko, O.; Jasicka-Misiak, I. Spectrophotometric and Chromatographic Assessment of Total Polyphenol and Flavonoid Content in Rhododendron Tomentosum Extracts and Their Antioxidant and Antimicrobial Activity. *Molecules* **2024**, *29* (5), 1095. https://doi.org/10.3390/molecules29051095.
